# Notoginsenoside R1 improves intestinal microvascular functioning in sepsis by targeting Drp1-mediated mitochondrial quality imbalance

**DOI:** 10.1080/13880209.2024.2318349

**Published:** 2024-02-22

**Authors:** Dongyao Hou, Ruixue Liu, Shuai Hao, Yong Dou, Guizhen Chen, Liangming Liu, Tao Li, Yunxing Cao, He Huang, Chenyang Duan

**Affiliations:** a Department of Anesthesiology, The Second Affiliated Hospital of Chongqing Medical University, Chongqing, P.R. China; b Research Institute of General Surgery, Jinling Hospital, Medical School of Nanjing University, Nanjing, P.R. China; c Department of Shock and Transfusion, State Key Laboratory of Trauma, Burns and Combined Injury, Daping Hospital, Army Medical University, Chongqing, P.R. China; d Department of Intensive Care Unit, The Second Affiliated Hospital of Chongqing Medical University, Chongqing, P.R. China

**Keywords:** Traditional Chinese medicine, mitochondria, sepsis, Drp1

## Abstract

**Context:**

Sepsis can result in critical organ failure, and notoginsenoside R1 (NGR1) offers mitochondrial protection.

**Objective:**

To determine whether NGR1 improves organ function and prognosis after sepsis by protecting mitochondrial quality.

**Materials and methods:**

A sepsis model was established in C57BL/6 mice using cecum ligation puncture (CLP) and an *in vitro* model with lipopolysaccharide (LPS, 10 µg/mL)-stimulated primary intestinal microvascular endothelial cells (IMVECs) and then determine NGR1’s safe dosage. Groups for each model were: *in vivo*—a control group, a CLP-induced sepsis group, and a CLP + NGR1 treatment group (30 mg/kg/d for 3 d); *in vitro*—a control group, a LPS-induced sepsis group, and a LPS + NGR1 treatment group (4 μM for 30 min). NGR1’s effects on survival, intestinal function, mitochondrial quality, and mitochondrial dynamic-related protein (Drp1) were evaluated.

**Results:**

Sepsis resulted in approximately 60% mortality within 7 days post-CLP, with significant reductions in intestinal microvascular perfusion and increases in vascular leakage. Severe mitochondrial quality imbalance was observed in IMVECs. NGR1 (IC_50_ is 854.1 μM at 30 min) targeted Drp1, inhibiting mitochondrial translocation, preventing mitochondrial fragmentation and restoring IMVEC morphology and function, thus protecting against intestinal barrier dysfunction, vascular permeability, microcirculatory flow, and improving sepsis prognosis.

**Discussion and conclusions:**

Drp1-mediated mitochondrial quality imbalance is a potential therapeutic target for sepsis. Small molecule natural drugs like NGR1 targeting Drp1 may offer new directions for organ protection following sepsis. Future research should focus on clinical trials to evaluate NGR1’s efficacy across various patient populations, potentially leading to novel treatments for sepsis.

## Introduction

Sepsis, which poses a major clinical challenge in acute and critical care, is commonly caused by pneumonia, abdominal infections, and urinary tract infections (Prescott and Angus 2018). Globally, over 19 million individuals are diagnosed with sepsis annually (Prescott and Angus 2018), with mortality rates of >25% (Reinhart et al. 2017). Among those who recover from sepsis, approximately three million subsequently develop cognitive impairment (Prescott and Angus 2018). Sepsis begins with infection and eventually causes cytokine storms, capillary endothelial damage, capillary leakage, microthrombosis, and decreased tissue perfusion, leading to organ dysfunction. Moreover, tissue hypoxia occurs because of microvascular dysfunction, which causes parenchymal cells to switch from aerobic to anaerobic respiration, producing toxic byproducts such as reactive oxygen species (ROS). ROS accumulation promotes cellular damage and endothelial cell dysfunction, which can perpetuate severe metabolic disorders at the tissue level, as well as sepsis-induced multiple-organ dysfunction (Lemasters and Nieminen 1997; Biesalski and McGregor 2007; Exline and Crouser 2008; Souza et al. 2015). Numerous studies have shown that normal microvascular function is crucial for maintaining intestinal barrier function (Haak and Wiersinga 2017), which is essential for combating sepsis (Saijo et al. 2015).

*Panax notoginseng* (Burk.) F.H.Chen (Araliaceae) is a traditional Chinese medicinal herb that is used to stop bleeding, prevent blood stasis, reduce swelling, and relieve pain. The whole or total saponin extracted from the plant is commonly used in medicinal preparations to treat cardiovascular and cerebrovascular diseases. The main active compound, notoginsenoside R1 (NGR1), which belongs to the protopanaxatriol group of *Panax notoginseng*, exerts therapeutic effects against allergic rhinitis (Zhang Y et al. 2022), diabetes (Zhang B et al. 2018, 2019; Zhou et al. 2019), osteoarthritis (Zhang et al. 2020), and cardiovascular diseases (Li et al. 2022; Xu et al. 2022). Furthermore, it can alleviate lung (Cao et al. 2021) and kidney injury (Shou et al. 2022) and improve cardiac function (Sun et al. 2013; Zhong et al. 2015) in sepsis owing to its anti-inflammatory effects. However, it remains unclear whether NGR1 protects microvascular function in patients with sepsis.

In recent years, the involvement of mitochondria in various disease processes has received considerable attention (Cloonan and Choi 2016; Chan 2020). Mitochondria play key roles in providing metabolites (Pietrocola et al. 2015) and regulating cell signalling (Raimundo 2014), calcium intake (Baughman et al. 2011; De Stefani et al. 2011), and cell death (Zorov et al. 2000; Bernardi and Di Lisa 2015). In our previous study, we found that mitochondrial dysfunction plays a crucial role in pathogenesis (Zhu et al. 2021), especially in severe sepsis and infectious shock, which have a poor prognosis and commonly lead to multiple-organ failure and death (Zhang H et al. 2018). Therefore, improving mitochondrial function in sepsis could protect several organs, including the brain, heart, lungs, liver, and kidneys, thereby improving disease prognosis (Chopra et al. 2011; Thomas et al. 2011). However, the mechanism of NGR1-mediated regulation of mitochondrial function in sepsis requires further investigation.

In order to gain a deeper understanding of the pathogenesis of sepsis, search for new treatment strategies and drug targets, and explore new perspectives on mitochondrial function in other diseases, our study investigates the protective effects of NGR1 on septic microvascular function and NGR1-mediated targeting of the mitochondrial dynamics-related protein (Drp1). We also evaluated the effects of NGR1 on Drp1 expression using septic mice and *in vitro* cell models. The models were constructed using cecum ligation puncture (CLP) of C57BL/6 mice and lipopolysaccharide (LPS) treatment of intestinal microvascular endothelial cells (IMVECs). Our results suggest that NGR1 may alleviate intestinal microvascular injury and improve prognosis after sepsis by inhibiting mitochondrial translocation of Drp1 and improving mitochondrial quality in IMVECs.

## Materials and methods

### Animals and materials

Male C57BL/6 mice, weighing 25-30 g and aged 9-10 weeks, were obtained from the Experimental Animal Center of Chongqing Medical University. The study protocol was approved by the Animal Care and Use Committee of Chongqing Medical University (CQMU-20220-222), and conformed to the guidelines of the National Institutes of Health Guide for the Care and Use of Laboratory Animals.

**Figure 1.**
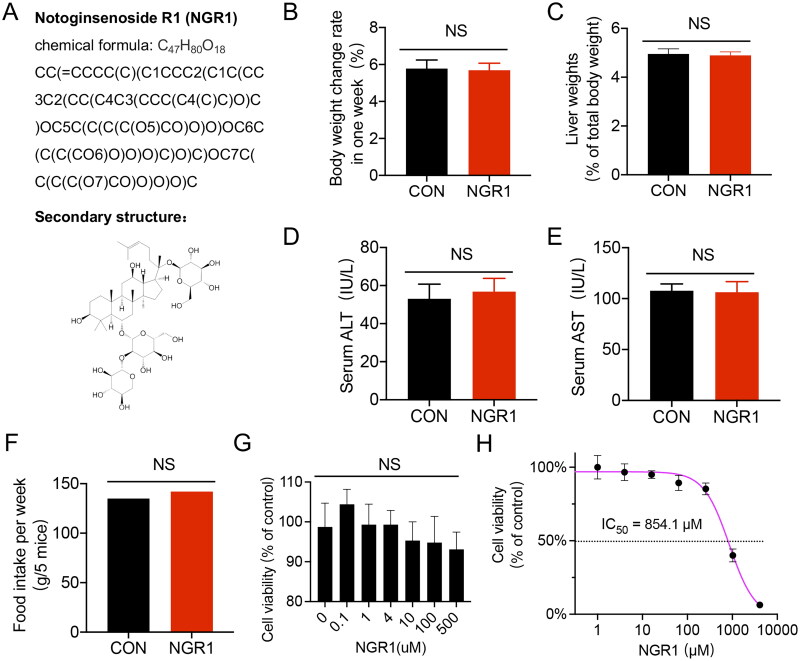
Structural information and safety assessment of NGR1. (A) Structural information of NGR1. (B-F) Different parameters of animal after receiving an intraperitoneal injection of NGR1 (30 mg/kg, once per day for 3 d) after 7 d. B: Body weight change rate (7 d). C: Liver weight (% of total body weight). D: Serum ALT levels. E: Serum AST levels. F: Food intake (g/5 mice after 7 d). (G,H) The cell viability of IMVECs treated with different concentrations of NGR1 for 30 min through different assays.G: MTT asay, H:CCK-8 assay. NS, *p >* 0.05 compared with the control group.

Notoginsenoside R1 (N3915) was purchased from Sigma-Aldrich (St. Louis, MO, USA). The structural information of NGR1 is provided in Figure 1(A). Antibodies against Drp1 (ab56788), β-actin (ab6276), and COX4 (ab110272) were purchased from Abcam (Cambridge, MA, USA). The β-tubulin (sc-5274) antibody was purchased from Santa Cruz Biotechnology (Santa Cruz, CA, USA). MitoTracker (Deep Red, M22426) was purchased from Invitrogen (Carlsbad, CA, USA). The JC-1 mitochondrial membrane potential fluorescent probe (ΔΨm) assay reagent (C2006), ROS assay working solution (S0033S), RIPA buffer (P0013B), protease inhibitor (P1045-1), phosphatase inhibitor (P1045-2), horseradish peroxidase-labelled goat anti-mouse IgG (H + L) (A0216), recombinant trypsin (P4201) and type II collagenase (ST2303) were purchased from Beyotime Biotechnology (Shanghai, China). Endothelial Cell Growth Supplement (ECGS) (E0760) was purchased from Millipore (Darmstadt, Germany). The cytoplasmic isolation kit (SC-003) and mitochondrial isolation kit (MP-007) were purchased from Invent Biotechnologies (Beijing, China). The Pro-Light HRP chemiluminescence detection reagent (PA112) was purchased from Tiangen Biotech (Beijing, China). A bicinchoninic acid (BCA) protein assay kit (23227) was purchased from Thermo Fisher Scientific (Waltham, MA, USA).

### Extraction and culture of primary IMVECs

Ten male C57BL/6 mice were anesthetized with pentobarbital (30 mg/kg) and euthanized by cervical dislocation, and the intestinal tissues (jejunum and ileum) were excised, sectioned longitudinally with a pair of ophthalmic scissors, and rinsed with ice-cold sterile phosphate buffered saline (PBS) containing 5% penicillin-streptomycin mixture. The intestinal tissues were then cut into 1 cm-long strips and stirred in 0.05% trypsin solution for 10 min before removing the epithelial cells at 37 °C. Tissue strips were cut into 1 mm-thick slices and digested in 2% type II collagenase solution at 37 °C for 15 min. After enzymatic digestion and mechanical homogenization, the suspension was filtered through a 400-mesh sieve. The filtrate was centrifuged at 1200 rpm for 5 min at 4 °C and the supernatant was discarded. The harvested cells were resuspended in IMVEC-complete medium (high-glucose DMEM + 20% foetal bovine serum, 0.05 mg/mL ECGS, and 1% penicillin-streptomycin mixture) and incubated at 37 °C under 5% CO_2_. After 4 h of cell adhesion, the medium- and slow-adherent cells were removed and cultured in the IMVEC-complete medium. Primary IMVECs were obtained from adherent cells. When the cells reached 80-85% confluence, they were rinsed twice with PBS, treated with 0.125% trypsin for 3 min, transferred to new culture flasks at a 1:2 dilution ratio, and cultured for 2-3 generations.

### Model preparation

The mice were acclimatized to 22 °C, 40-70% humidity, and a 12 h light/dark cycle and fed pelleted rodent standard mouse food and filtered water *ad libitum*. Before the experiment (after 1-week adaptation), the food supply was restricted, but the mice were allowed to drink water freely. To replicate the sepsis model *via* CLP (Rittirsch et al. 2009), mice were initially anesthetized with pentobarbital (30 mg/kg) until they did not respond to needle stimulation. During the experiment, the body temperature was maintained at 36-38 °C using a heating pad, and all procedures were performed under sterile conditions. The skin was incised at the ventral midline and 50% of the cecum was ligated with a 4.0 wire below the ileocecal flap. Subsequently, a small amount of the content was expelled from the abdominal cavity using a 21 gauge needle that punctured the ligated portion of the cecum. Thereafter, the cecum was restored to its original position in the abdomen and the wound was closed using simple running sutures to the abdominal musculature and skin. After closing the abdomen, the mice were returned to their cages and subcutaneously injected with 50 mL/kg of saline at 37 °C for fluid resuscitation. After completing the CLP procedure, the mice were allowed to drink and eat freely and were subcutaneously injected with 0.05 mg/kg buprenorphine every 6 h for 2 d to induce postoperative analgesia. For the *in vitro* experiments, LPS (10 µg/mL) was used to induce IMVECs for 12 h to simulate cellular-level sepsis.

### Cell viablity detection

MTT and CCK-8 assays were conducted to detect cell viability based on the instructions. Briefly, cells were first evenly inoculated in a 96-well plate (2 × 10^3^/well), placed in a 5% CO_2_ incubator at 37 °C, and then cultivated until the monolayer of the cells filled the bottom of the hole. Next, 0.1, 4, 10, 100, 500 μM NGR1 was added for the MTT assay and 1, 4, 16, 64, 256, 1024, 4096 μM NGR1 was added for the CCK-8 assay, and then cells were incubated for 30 min. A 10 μL MTT solution (5 mg/mL) or a 10 μL CCK-8 solution was then added to each well and the cells were allowed to continue to incubate for 4 h. For the MTT assay, the supernatant was removed and the crystals were dissolved in 150 μL DMSO. Absorbance was measured with a multimode reader (Spark; TECAN, Mannedorf, Switzerland) at a test wavelength of 490 nm. For the CCK-8 assay, the absorbance was measured with a multimode reader at 450 nm, and the IC_50_ value was then calculated based on the cell viability curve.

**Figure 2.**
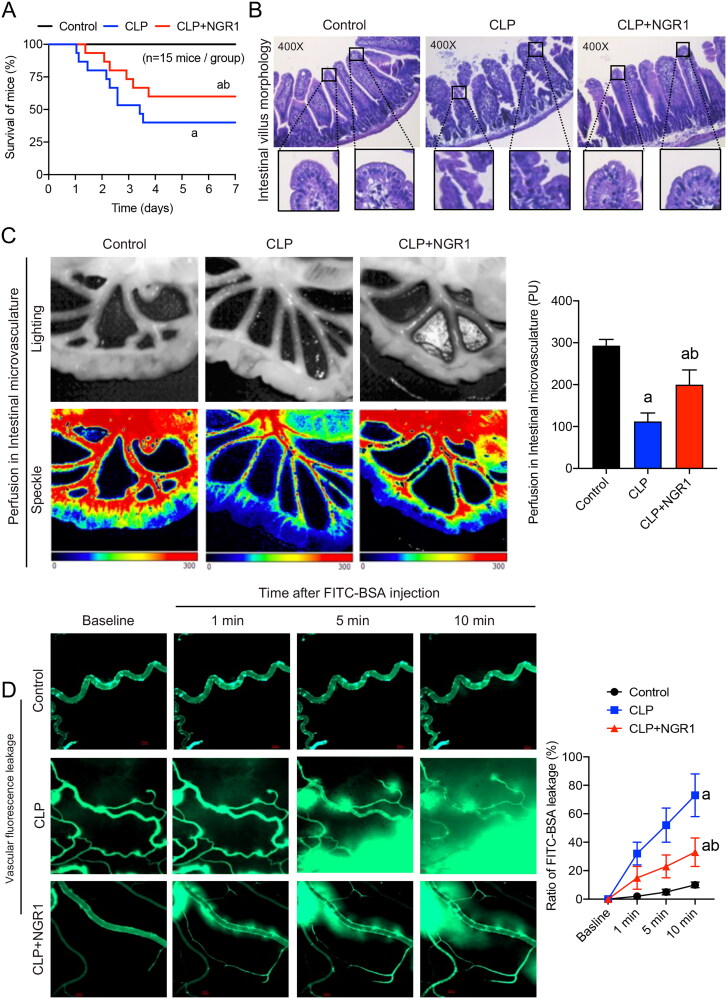
Effects of NGR1 on survival and intestinal function of septic mice. (A) Survival rate and survival time (*n* = 15). (B) HE staining of intestinal villi to observe gross morphology (400×). (C) Speckle tomography images of mesenteric perfusion and related statistics. (D) FITC-BSA leakage of mesenteric microveins (*n* = 8). Data are presented as mean ± standard deviation. a, *p* < 0.05 compared with the control group; b, *p* < 0.05 compared with the CLP group.

### Treatment and group design

The mice were randomly divided into three groups (15 mice per group): control, sepsis (CLP; established as described above), and treatment (CLP + NGR1). The cecum in the control group was ligated with anaesthetic but without puncturing, and the rest of the procedure was the same as that described above. NGR1 (30 mg/kg, intraperitoneal injection, once per day for 3 d) or saline of the same volume was administered postoperatively to the treatment and control groups, respectively. The upper limit of the statistical survival time was 7 d. The remaining mice were divided into the same groups and used for sampling and observation of mesenteric blood flow and intestinal villus morphology. IMVECs were treated with LPS (10 µg/mL) to simulate a septic state at the cellular level. LPS-induced IMVECs were treated with NGR1 (4 μM) for 30 min.

### Haematoxylin and eosin (HE) staining of the intestinal microvilli

The mice were anesthetized and euthanized by cervical dislocation. Fresh colonic tissue was fixed in formalin and embedded in paraffin according to standard pathological practice. Standard sections (3–4 µm thick) were cut under a microscope to obtain optimal orientation and subjected to HE staining. Subsequent observation of intestinal microvilli morphology (400×) was performed using a microscope (Leica DM2500; Leica Microsystems, Wetzlar, Germany), as previously described (Duan et al. 2020a).

### Speckle tomography of mesenteric perfusion

Mesenteric blood flow was measured using a Doppler imager (Peri-Cam PSI-ZR; Perimed, Stockholm, Sweden), with the mesentery exposed to a laser at a distance of 14 cm. Color images were used to indicate the relative perfusion levels at specific points, and mesenteric perfusion was analysed using PIMsoft software (Perimed, Stockholm, Sweden), as previously described (Duan et al. 2020c; Zeng et al. 2022).

### Measurement of mesenteric microvein permeability

The mesenteric ileocecal region was exposed and placed on a transparent slide moistened with saline at 37 °C to maintain appropriate temperature and humidity. Images were captured using an ultrasensitive camera (HAMAMATSU, Hamamatsu, Japan) attached to a microscope. After 10 min of basal observation, 10 mg/kg FITC-BSA was intravenously injected and vascular fluorescence leakage was recorded for 10 min. The rate of change in mesenteric microvein permeability in mice was determined using Image-Pro Plus software (version 5.0; Media Cybernetics, Silver Springs, MD, USA).

### Mitochondrial morphology observation

IMVECs were seeded on confocal culture plates at a density of 1 × 10^5^ cells/well and cultured for approximately 2 d for confocal imaging. The mitochondrial morphology was observed as previously described (Zhang T et al. 2022). MitoTracker (Deep Red, 100 nM) was added and the cells were incubated for 30 min. Subsequently, mitochondria were observed using an inverted confocal microscope (Leica TCS SP5; Leica Microsystems) with a 60 × 1:3 NA oil immersion objective. Red fluorescence was excited using a 633 nm laser, and the emission spectra were obtained at 655-670 nm. Image-Pro Plus software was used to measure and compute the mitochondrial length and aspect ratios.

### Measurement of intracellular ROS

The intracellular ROS levels were measured as previously described (Zeng et al. 2022). The ROS assay working solution was added to the cells, which were then incubated for 30 min. The cells were imaged using Leica TCS SP5. Green fluorescence was excited with a 488 nm laser, and emission spectra were obtained at 501-563 nm. Images were analysed using the Image-Pro Plus software.

### Measurement of mitochondrial membrane potential (ΔΨm)

Mitochondrial membrane potential was measured as previously described (Duan et al. 2020b). The JC-1 staining working solution was added to the cells, which were then incubated for 30 min. The cells were imaged using Leica TCS SP5. The monomer was excited with a 488 nm laser, and emission spectra were obtained at 501-563 nm. Aggregate fluorescence was excited with a 633 nm laser, and emission spectra were obtained at 558-617 nm. Images were analysed using the Image-Pro Plus software.

### Subcellular fractionation

IMVECs were collected in centrifuge tubes and the cytoplasm and mitochondria were separated using cytoplasmic and mitochondrial separation kits (Duan et al. 2021), respectively, according to the manufacturer’s instructions. Isolated sub-cells were used for western blot analysis.

### Western blotting

Cells and sub-cells were lysed with RIPA buffer, and protease and phosphatase inhibitors were added. The cell samples were then electrophoresed, and the resolved proteins were blotted onto polyvinylidene fluoride membranes. The membranes were then incubated with Drp1 primary antibody and then with horseradish peroxidase-labelled secondary antibodies. Protein concentrations were calculated using a BCA protein assay kit. The blotted proteins were visualized using Pro-Light HRP chemiluminescence detection reagent. Band intensities were analysed using Quantity One software (version 4.62; Bio-Rad, Life Science, Hercules, CA, USA). β-actin, COX4, and β-tubulin were used as internal references for the total protein, mitochondrial, and cytoplasmic components, respectively. The ratio of Drp1 to the grayscale value of the internal reference was calculated using Image-Pro Plus and was assumed to represent the relative expression of the target protein.

### Small molecule microarray screening of Drp1-targeting drugs

We constructed Drp1 recombinant proteins and a small-molecule microarray chip containing over 3000 small-molecule active monomers for high-throughput screening of FDA-approved drugs, traditional Chinese medicine monomers, and small-molecule inhibitors that potentially target Drp1. Briefly, chips were added to the blocking solution and incubated at 25 °C for 1 h. After cleaning, samples containing biotin-labelled Drp1 and biotin-labelled 6× His-tag protein chips were added to the incubation solution (5 μg/mL) and incubated at 25 °C for 1 h. The chips were subsequently cleaned, incubated with the Cy5-SA incubation solution at 25 °C for 1 h in the dark, and cleaned again. Finally, the chips were placed in a chip dryer (Capitalbio 120090; Beijing, China), centrifugally dried, and scanned using a microarray-chip scanner (Capitalbio LuxscanTM 10 Detoxy-A) for data extraction. Raw data were obtained using GenePix Pro software (version 6.0; Molecular Devices, Sunnyvale, CA, USA) and analysed for potential small molecules. A fold change of ≥1.5 indicates candidate small molecules, and a P value of <0.05 indicated significant differences between candidates.

### Homology modelling and molecular docking

The structural formula of NGR1 was derived from the National Center for Biotechnology Information (NCBI) (https://pubchem.ncbi.nlm.nih.gov/compound/441934), and the molecular docking structures of NGR1 and Drp1 were generated using H-docking (Yan et al. 2017). The input sequence or structural transformation sequence was used to conduct a sequence similarity search on the Protein Data Bank (PDB) database to identify homologous sequences of the receptor and ligand molecules; two sets of homologous templates were generated. Subsequently, by comparing the two sets of templates to confirm whether they had a common record with the same PDB code, the appropriate template was selected, and a model was built using MODELELLER (Martí-Renom et al. 2000). Sequence alignment was performed using ClustalW software (Martí-Renom et al. 2000; Sievers et al. 2011). Finally, conventional global docking was performed using the server, and the optimal docking model was selected for plotting according to the scores and active sites.

### Statistical analysis

Statistical analyses were performed using the SPSS software (version 18.0; SPSS Inc., Chicago, IL, USA). Data are expressed as the mean ± standard deviation. Variance analysis was used to compare differences between groups, followed by minimum significant difference tests. Survival analysis was performed using the Kaplan–Meier method and calculated using SPSS (version 17.0). Statistical significance was set at *p*** **<** **0.05.

## Results

### Evaluation of the safety of NGR1 in mice and its effect on IMVECs viability

To investigate whether the dose of NGR1 (30 mg/kg, intraperitoneal injection, once per day for 3 d) induce toxicity or side effects in normal mice, we measured relevant indicators after 7 d. Our results revealed that administration of NGR1 to normal mice did not significantly alter the body weight change rate, liver weight, serum ALT, serum AST, food intake levels (*p*** **>** **0.05, Figure 1(B–F)). Furthermore, to test the influence of NGR1 on the viability of IMVECs, the cells were treated with increasing doses (0 μM-4096 μM) of NGR1 for 30 min. The results show only when the dose was under 10 μM, the cell viability remained at a relatively stable level (about 100%), and the IC_50_ was 854.1 μM (Figure 1(G, H)). Since Zhang Y et al. (2022] showed that the dosage of NGR1 in mice (30 mg/kg/d) and epithelial cells (4 μM) can reduce the mitochondrial fission in allergic rhinitis, the same dose was chosen by us to explore the effects of NRG1 on the mitochondria of endothelial cells under sepsis.

### Effects of NGR1 on survival, intestinal barrier function, and microvascular function in septic mice

We used the CLP method to simulate sepsis in mice. Half of the sepsis group mice died within 3 d after the procedure, and approximately 40% survived for more than 7 d, with a mean survival time of 4.1 d. Treatment with NGR1 increased the survival time of septic mice, with nearly three-quarters surviving for more than 3 d and approximately 60% surviving for more than 7 d, prolonging the mean survival time to 5.2 d (*p*** **<** **0.05, Figure 2(A)). These results indicated that intraperitoneal injection of NGR1 can significantly improve the survival rate of septic mice, thereby validating the clinical use of NGR1 in septic animals. HE staining showed that NGR1 treatment could alleviate sepsis-induced villous oedema, inflammatory infiltration, and epithelial mucosal detachment (Figure 1(B)). Furthermore, speckle tomography images showed that mesenteric blood flow was significantly reduced in the CLP group (*p*** **<** **0.05), whereas NGR1 treatment significantly improved the perfusion of intestinal microvasculature (*p*** **<** **0.05, Figure 2(C)). The results of the vascular fluorescence leakage assay showed that the FITC-BSA penetration rate in mesenteric microveins in the CLP group was significantly increased (up to 7-fold) compared with that in the control group at 10 min (*p*** **<** **0.05) and that mesenteric microvein exudation was significantly reduced after NGR1 treatment (*p*** **<** **0.05, Figure 2(D)). These results suggest that NGR1 significantly improves the survival rate of septic mice by effectively protecting the intestinal barrier and mesenteric vascular functions.

#### Effects of NGR1 on mitochondrial quality in LPS-induced IMVECs

**Figure 3.**
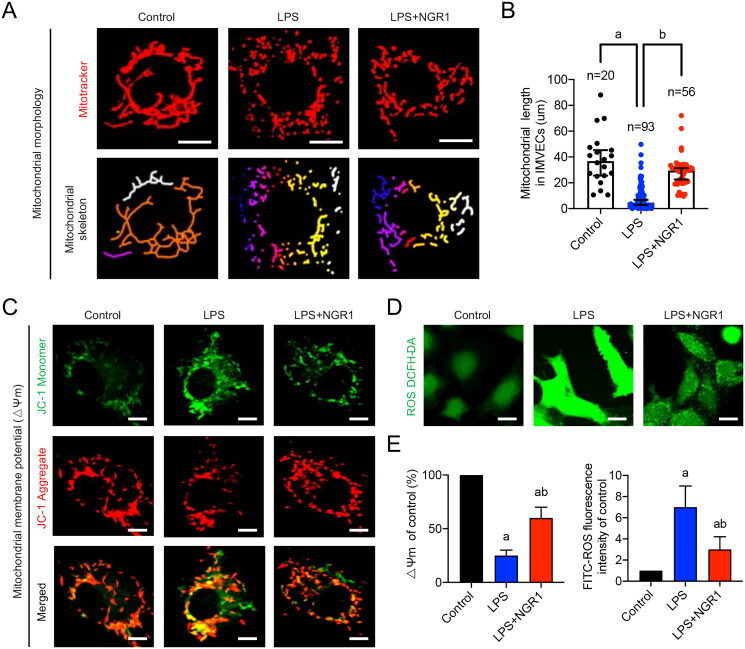
Effects of NGR1 on mitochondrial quality in LPS-induced IMVECs. (A) Representative confocal images of IMVEC mitochondrial morphology in each group (bar = 25 μm) and analysis of mitochondrial skeletons using Image J software. (B) Representative confocal images of mitochondrial membrane potential (ΔΨm) of IMVECs, which were labeled with JC-1 monomer (green fluorescent probe) and JC-1 aggregate (red fluorescent probe) (bar = 25 μm). (C) Representative confocal images of ROS fluorescence intensity in IMVECs (bar = 50 μm). (D) Statistical analysis of ΔΨm and ROS in IMVECs (*n* = 5). Data are presented as mean ± standard deviation. a, *p* < 0.05 compared with the control group; b, *p* < 0.05 compared with the LPS group.

To verify whether NGR1 affects mitochondrial quality in IMVECs under septic conditions, we stimulated IMVECs with LPS and evaluated several mitochondrial quality evaluation indices at the cellular level. First, we explored whether NGR1 could protect the mitochondrial morphology in LPS-induced IMVECs. Confocal microscopy images showed that the mitochondria in IMVECs were mainly characterized by long columns and reticules in the control group, whereas those in the LPS group exhibited fragmentation (*p*** **<** **0.05). After NGR1 treatment, the mitochondrial morphology was significantly improved (*p*** **<** **0.05, Figure 3(A)). Statistical analysis of mitochondrial length in IMVECs revealed that the median length in the control group was 36.825 μm, which was reduced to 4.400 μm in the LPS group and increased to 29.480 μm in the LPS + NGR1 group (*p*** **<** **0.05). In addition, the number of punctate mitochondria increased in LPS-induced IMVECs compared to that in the control group (*p*** **<** **0.05); however, this number decreased after NGR1 treatment (*p*** **<** **0.05, Figure 3(B)). These findings confirm that NGR1 treatment alleviates mitochondrial fragmentation in IMVECs after sepsis.

Furthermore, we evaluated the mitochondrial ΔΨm and ROS production in IMVECs. The confocal images revealed that the ΔΨm was reduced by 75% after LPS stimulation (*p*** **<** **0.05, Figure 3(C)), and ROS production increased 6-fold in LPS-induced IMVECs (*p*** **<** **0.05, Figure 3(D)); however, after NGR1 treatment, both values improved significantly (*p*** **<** **0.05, Figure 3(C–E)). These results suggest that NGR1 improves mitochondrial function in IMVECs under septic conditions, and its positive effects on sepsis-induced intestinal microvascular dysfunction may be closely related to the improvement of mitochondrial quality imbalance in IMVECs.

### Targeting effect of NGR1 on mitochondrial Drp1 in LPS-induced IMVECs

**Figure 4.**
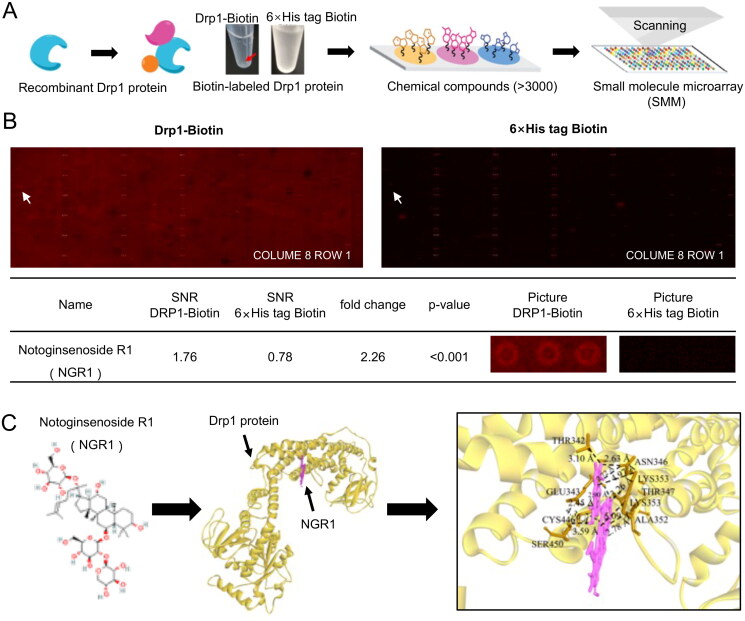
Targeting effect of NGR1 on Drp1, verified using small-molecule microarray chips. (A) Flow chart of small-molecule microarray chips used for screening interactions with Drp1. (B) Display of chip results. From left to right are the chip scan of experimental sample Drp1-biotin and global chip scan of control sample 6× His-tag biotin; chip results are analysed in the table below. (C) From left to right, the structural formula of NGR1, overall structural diagram of NGR1 docking to Drp1 (yellow portion is Drp1, pink portion is small molecule NGR1), and local diagram of the specific interaction sites.

As a classic mitochondrial dynamic-related protein, Drp1 primarily affects mitochondrial quality by regulating mitochondrial fission (Duan et al. 2020b). We compared the small-molecule compounds bound to the biotin-labelled Drp1 chip and biotin-labelled 6× His-tag protein chip (negative control) (Figure 4(A)) and found that NGR1 may specifically physically bind to recombinant Drp1 (fold change = 2.26, *p*** **<** **0.001; Figure 4(B)). These findings suggest that NGR1 may preserve mitochondrial quality by targeting Drp1. To further verify the results of the small-molecule microarray experiment, we generated homology and molecular docking models of Drp1 and NGR1. The results showed that NGR1 could directly bind to Drp1 in a compact conformation and interact with the amino acids surrounding Drp1 (Figure 4(C)).

**Figure 5.**
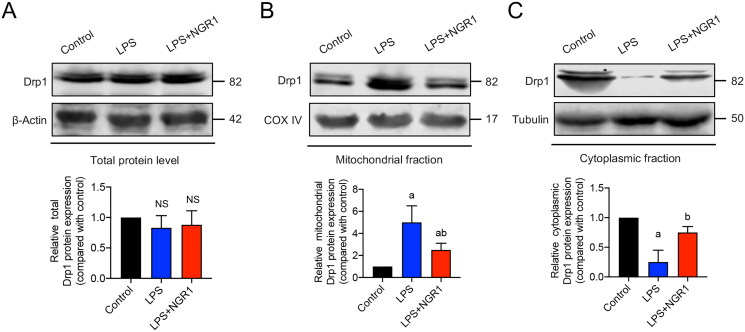
Effects of NGR1 on Drp1 expression in subcellular fractions of IMVECs. Western blotting results showing the effect of NGR1 treatment on the expression of (A) total Drp1, (B) mitochondrial Drp1, and (C) cytoplasmic Drp1 in IMVECs under sepsis (*n* = 5). Data are presented as mean ± standard deviation. a, *p* < 0.05 compared with the control group, b, *p* < 0.05 compared with the LPS group. NS, *p >* 0.05 compared with the control group.

To elucidate the specific mechanism by which NGR1 regulates Drp1, we measured Drp1 expression in different IMVEV subcellular fractions. Western blotting results showed that there were no significant differences in total Drp1 expression between the LPS group and control group (*p*** **>** **0.05, Figure 5(A)). However, compared with the control group, in the LPS group, the expression of mitochondrial Drp1 was significantly increased (*p*** **<** **0.05, Figure 5(B)), whereas that of cytoplasmic Drp1 was significantly decreased (*p*** **<** **0.05, Figure 5(C)). This finding suggests that Drp1 translocates from the cytoplasm to the mitochondria in LPS-induced IMVECs. The translocation was significantly reduced in the NGR1 treatment group (*p*** **<** **0.05, Figure 5) compared with that in the LPS group. These results suggest that NGR1 may improve mitochondrial quality in IMVECs under septic conditions by targeting Drp1 and preventing its translocation to mitochondria.

## Discussion

Several studies have demonstrated that improving the intestinal barrier and alleviating microvascular dysfunction are crucial for sepsis treatment (Vincent et al. 2013; Saijo et al. 2015; Schmidt et al. 2015; Haak and Wiersinga 2017). Various recently discovered mechanisms of action of traditional Chinese medicines and their influence on disease processes have received widespread attention (Parekh et al. 2009; Hao et al. 2017; Liu et al. 2018; Huang et al. 2021). *Panax notoginseng* is used in traditional Chinese medicine to prevent blood stasis, reduce swelling, and alleviate pain. It is often used to treat cardiovascular and cerebrovascular diseases. NGR1 has been found to prevent vital organ damage in sepsis mainly by inhibiting inflammatory cytokines (Sun et al. 2013; Cao et al. 2021). In this study, the safe doses of NGR1 on mice (30 mg/kg/d) and mouse intestinal microvascular endothelial cells (4 μM) were determined through experiments and relevant literature, and we investigated the protective effects of NGR1 on the intestinal barrier and microvascular function after sepsis and further analysed the possible regulatory mechanisms underlying these effects.

The feedback cycle of mitochondrial dynamics ensures a mitochondrial quality balance under physiological conditions (Wu et al. 2017), this process is closely associated with oxidative stress and is vital for preserving mitochondrial function and homeostasis (Calabrese et al. 2010). Disruption of mitochondrial dynamics may induce mitochondrial dysfunction, metabolic disorders, and mitophagy, all of which can lead to the development of serious diseases, such as neurodegenerative syndrome (Calabrese et al. 2007; Soreq et al. 2012), pulmonary arterial hypertension (Marsboom et al. 2012), and aortic valve stenosis (Liu C et al. 2022). Pathological stimuli, such as ischemia, hypoxia (Zeng et al. 2022), infection, and inflammation (Duan et al. 2022) may lead to mitochondrial fragmentation, resulting in decreased ATP production, disrupted calcium metabolism, and eventually, mitochondrial dysfunction. Mitochondrial dysfunction plays a crucial role in the pathogenesis of sepsis, particularly severe sepsis, and is a major contributing factor to poor prognosis, multiple-organ failure, and death (Zhang H et al. 2018).

Extensive literature suggests that enhancing mitochondrial function and regulating oxidative stress can provide cellular protection (Calabrese et al. 2009; Siracusa et al. 2020). NGR1, a traditional Chinese medicine, has garnered significant attention for its haemostatic and anti-inflammatory properties. In addition, NGR1 is known to protects the mitochondria. Zhou et al. (2019) found that NGR1 ameliorated diabetic retinopathy through Pink1-dependent activation during mitophagy. Additionally, Liu B et al. (2022) found that NGR1 alleviates mitochondrial dysfunction, thereby preventing neuronal energy failure during acute focal cerebral ischemia. Our study showed that NGR1 effectively prevents excessive sepsis-induced mitochondrial fission and significantly improves mitochondrial function.

Drp1 plays an important role in regulating the mitochondrial dynamics. Under physiological conditions, Drp1 is primarily distributed in its polymeric form in the cytoplasm. After activation, Drp1 translocates from the cytoplasm to the outer mitochondrial membrane and polymerizes to form a ring structure, which causes mitochondrial fission (Ji et al. 2017). We confirmed the specific binding between NGR1 and Drp1 using small-molecule microarray chips and molecular docking. Small-molecule microarray chips are novel tools, and the microarray chip used in the present study contained approximately 1000 Traditional Chinese Medicine (TCM) monomers, 1500 FDA-approved drugs, 800 small-molecule inhibitors, and over 3000 total small-molecule active monomers. The chip is suitable for studying the interactions between pure/total proteins and small molecules (Calabrese et al. 2018), nucleic acids, and small molecules (Abulwerdi et al. 2019). It is not only a powerful tool for small-molecule probe discovery but is also rapidly becoming a platform for high-throughput proteomic analysis. Recently, Zhang Y et al. (2022) found that NGR1 attenuates allergic rhinitis through AMPK/Drp1-mediated mitochondrial fission, which is consistent with our findings. We further confirmed that NGR1 targets Drp1 and can reduce its translocation of Drp1 from the cytoplasm to the mitochondria, thereby inhibiting excessive mitochondrial fission and preserving mitochondrial function in IMVECs after sepsis.

Although our experiments suggest that NGR1 ameliorates intestinal microvascular damage by inhibiting Drp1-mediated mitochondrial fission, there were some limitations that require further investigation. (1) In this study, we verified the protective effects of NGR1 in a mouse CLP model and in LPS-induced IMVECs. However, this animal model simulated moderate sepsis and could not accurately simulate acute fatal sepsis or the complex pathophysiological environment of the human body. Therefore, it is unclear whether NGR1 exerts similar protective effects under these conditions. (2) As the impairment of vascular endothelial barrier function is a key pathophysiological process in sepsis development (Koh et al. 2010; Armstrong et al. 2013), we focused on the effects of NGR1 on the intestinal barrier and vascular endothelial cells. However, it is unknown whether NGR1 has a protective effect on mitochondria in other important organs such as the heart, liver, and kidneys. In addition, we found a potential binding target effect of NGR1 on Drp1, this finding provides impetus for the development of new treatment strategies, drug design, or clinical trials, it also offers a new perspective for researchers to explore the role of mitochondria in other diseases or inflammatory states. However, elucidating the specific targeting sites will require further investigation *via* point mutation experiments.

## Conclusions

Our research demonstrated that sepsis leads to significant intestinal barrier and microvascular dysfunction. By targeting the mitochondrial translocation of Drp1, NGR1 treatment was found to enhance mitochondrial quality in IMVECs, resulting in improved intestinal microvascular function. The data suggest that targeting Drp1-mediated mitochondrial quality imbalance presents a viable therapeutic approach in sepsis management. NGR1, a natural small molecule drug that inhibits Drp1, emerges as a promising strategy for organ protection following sepsis. These findings underscore the potential of Drp1 as a therapeutic target and highlight NGR1’s role in mitigating sepsis-induced damage. Future investigations should aim at conducting clinical trials to verify NGR1’s efficacy in a diverse range of patients with sepsis, potentially broadening the treatment options for this condition.

## Data Availability

The datasets of this study can be found at https://www.jianguoyun.com/p/DVeURM0QtMvLChjY0fUEIAA.
